# A Global Survey on Opioid Stewardship Practices in Hospitals: A Cross-Sectional Pilot Study

**DOI:** 10.3390/pharmacy9030122

**Published:** 2021-07-01

**Authors:** Sarah Al-Samawy, Nisha Varughese, Regis Vaillancourt, Xiao Yu (William) Wang, Jonathan Penm

**Affiliations:** 1 School of Pharmacy and Medical Sciences, University of South Australia, City East Campus, 101 Currie St, Adelaide, SA 5001, Australia; sarahalsamawy98@gmail.com; 2 Children’s Hospital of Eastern Ontario, 401 Smyth Rd, Ottawa, ON K1H 8L1, Canada; nvarughese@cheo.on.ca (N.V.); rvaillancourt@cheo.on.ca (R.V.); 3 Mount Sinai Hospital, 600 University Ave, Toronto, ON M5G 1x5, Canada; william.wangrx@hotmail.com; 4 Faculty of Medicine and Health, School of Pharmacy, The University of Sydney, Pharmacy and Bank Building A15, Science Rd, Camperdown, NSW 2006, Australia; 5 Department of Pharmacy, Prince of Wales Hospital, 320-346 Barker St, Randwick, NSW 2031, Australia

**Keywords:** opioid, prescribing, appropriate, pharmacists, survey, hospitals

## Abstract

Objective: The objectives of this study are to describe opioid stewardship practices in hospitals being implemented globally, in addition to investigating the attitudes and perceptions of health professionals regarding opioid stewardship in the hospital setting. Methods: A survey was developed by the research team to ask about participants’ attitudes and perceptions regarding opioid stewardship practices. The survey was piloted for performance by five independent third-party healthcare professionals prior to being made available online, being hosted using Research Electronic Data Capture software, with invitations distributed by the International Pharmaceutical Federation (FIP). Descriptive analyses were used to describe the features of the study, and responses obtained from the survey were further categorised into subgroups separating answers relating to attitudes and perceptions, and policies and regulations. Results: Overall, there were 50 respondents from 18 countries, representing an 8% response rate from the FIP hospital pharmacy section mailing list. In total, 33/50 (66%) participants agreed opioids are overused nationally, with 22/49 (45%) agreeing they are overused at their workplace. Furthermore, 32/50 (64%) agreed the opioid crisis is a significant problem nationally, and 44/50 (88%) agreed opioid stewardship would reduce problems associated with the opioid crisis. Policies to educate providers about safe opioid prescribing were uncommon, not exhibited in 26/46 (57%) of hospitals, with all EMR and SE Asia hospitals not displaying this policy. Policy for investigation of narcotic discrepancies was present in 34/46 (74%) of hospitals, and there was a policy for reporting discrepancies at 33/46 (72%) hospitals. Conclusion: In conclusion, healthcare professionals in the American region are more likely to perceive the opioid crisis as a problem, as opposed to those from the European region. Regardless of the presence or absence of a crisis, the implementation of further opioid education and stewardship practices are necessary globally and will contribute to safer prescribing and utilisation practices in hospitals.

## 1. Introduction

The opioid crisis appears to be occurring globally with reports from the United States, United Kingdom, Canada, and Australia stating a high prevalence of opioid-related adverse events (ORAEs) and deaths. Prescription opioids appear to be a large contributor to opioid overdoses and occur primarily unintentionally in patients [1]. ORAEs can also include respiratory depression, immunosuppression, physical dependence and withdrawal effects, and death.

According to the World Health Organisation (WHO), the number of opioid overdoses has been increasing in multiple countries, and this is partly attributable to the increasing use of opioids for conditions such as chronic pain management. WHO reports that globally, approximately half a million deaths are attributable to drug use, and of these deaths, over 70% are opioid related, with over 30% resulting from overdose [2], indicating the extent and severity of the potential consequences of opioids.

Hospitals are significant contributors to prescription opioids, being an environment whereby initial opioid use often occurs [3]. One study showed that of 1.14 million non-surgical hospital admissions in the US, 51% received opioids, of which 52% received opioids on the day of discharge [4]. Additionally, another study displayed that prescribing opioids to opioid-naïve patients at discharge was associated with almost five times increased odds of chronic opioid use one year post discharge (adjusted OR = 4.9, 95% CI 3.22–7.45) [5]. Hence, opioid prescribing in hospitals is a large contributor to chronic opioid use.

Overuse of opioids in hospitals contributes to chronic opioid use by patients and is also responsible for a large proportion of adverse drug events that occur during a patient’s hospital admission. One study established the association between hospital opioid-prescribing rates, showing hospitals with high opioid-prescribing rates (opioid exposure 62% with 0.39% ORAEs) have almost twice the rate of ORAEs, as opposed to hospitals with low opioid-prescribing rates (opioid exposure 38% with 0.21% ORAEs) [4]. Furthermore, the occurrence of opioid overdoses in the hospital setting is a significant but preventable issue. A review of 13 hospitals in the US and the United Kingdom determined that of over 19 million inpatients, 0.06–2.50% of hospitalisations resulted in opioid overdose, and these results are likely higher than reported previously due to a lack of detection methods [6], emphasising the importance of appropriate opioid use in hospitals to prevent ORAEs and overdoses.

Opioid stewardship aims to promote safe and appropriate opioid-prescribing practices and utilisation in hospitals, preventing adverse events. Common methods utilised in opioid stewardship programs include promoting the utilisation of adjunct nonopioid analgesia such as paracetamol and nonsteroidal anti-inflammatories, limiting the prescribing quantities of opioids, incorporating pharmacist counselling regarding appropriate use, handling, and disposal of opioids, and educating junior staff members and other prescribers [7]. A review of opioid stewardship practices showed that academic detailing reduced prescription errors from 41% to 24% [8] and increased adherence of prescribed opioids to predetermined recommendations, whilst education strategies reported 79.0% to 95.9% increased nonopioid analgesic use [8]. As opioid stewardship practices have been effective in improving opioid use in hospital settings, it is important to monitor the implementation of these practices to identify areas of improvement and share success stories. A national survey of hospital pharmacy practice conducted by the American Society of Health-System Pharmacists (ASHP) in 2019 found that 47.3% of hospitals surveyed had implemented opioid stewardship methods [9]. Another survey in Australia and New Zealand showed that among 45 respondents, there was significant variability in opioid stewardship measures applied and the extent of their implementation, with one hospital reporting having no measures in place, and other hospitals having up to nine opioid stewardship measures implemented [10]. Due to the extensive variations in practice, it is unclear which strategies are difficult to improve or may be country specific. Conducting a global survey of opioid stewardship practices will help determine which strategies could be applied globally and which ones need local adaptation, hence the aim of this pilot study.

## 2. Objectives

The objectives of this study are to describe opioid stewardship practices in hospitals being implemented globally, in addition to investigating the attitudes and perceptions of health professionals regarding opioid stewardship in the hospital setting.

## 3. Methods

### 3.1. Survey Development

A survey was developed by the research team and asked about the presence of policies and regulations regarding the clinical use, prescribing, and destruction of opioids in hospitals. Participants were also surveyed regarding their attitudes and perceptions towards opioid stewardship practices. These questions asked participants to rank strategies for appropriate opioid use in order of importance and the outcomes of the practices.

Additionally, they were asked to complete a five-point Likert scale on their level of agreement towards current opioid use, stewardship practices, and the presence of an opioid crisis in their institution and country. Lastly, participants were asked to complete demographic questions related to themselves and their institution. The survey was piloted for performance by five independent third-party healthcare professionals prior to being made available online (Appendix A).

### 3.2. Survey Distribution

The survey was hosted online using Research Electronic Data Capture (REDCap, Elsevier, Amsterdam, The Netherlands) software [11]. An invitation to complete the survey was distributed by the International Pharmaceutical Federation (FIP) Hospital Pharmacy Section via email. The invitation was sent out between February and March 2020. Following the invitation, reminder e-mails were sent at 1 week and 3 weeks. After 4 weeks, the survey was closed. Participation was voluntary, and no compensation was offered.

### 3.3. Data Analysis

Responses were presented for the institution, with individuals asked to respond on behalf of their hospital. Hence, no hospitals had more than 1 respondent. Descriptive analyses were used to describe the features of the study; descriptive data were analysed and categorised according to regions using the World Health Organisation’s (WHO) classification [12]. Eastern Mediterranean (EMR) and South East (SE) Asia were combined due to the low number of responses from these regions. Agreement to survey items was compared by region using Fisher’s exact test. A two-tailed *p*-value below 0.05 was considered statistically significant. All statistical analyses were performed using IBM SPSS Statistics Version 25 (IBM, Armonk, NY, USA).

## 4. Results

Out of the 653 surveys distributed, there were 50 respondents to the survey from 18 countries, representing an 8% response rate from the FIP hospital pharmacy section mailing list, the demographics of these respondents can be observed in Table 1.

**Figure 1:**
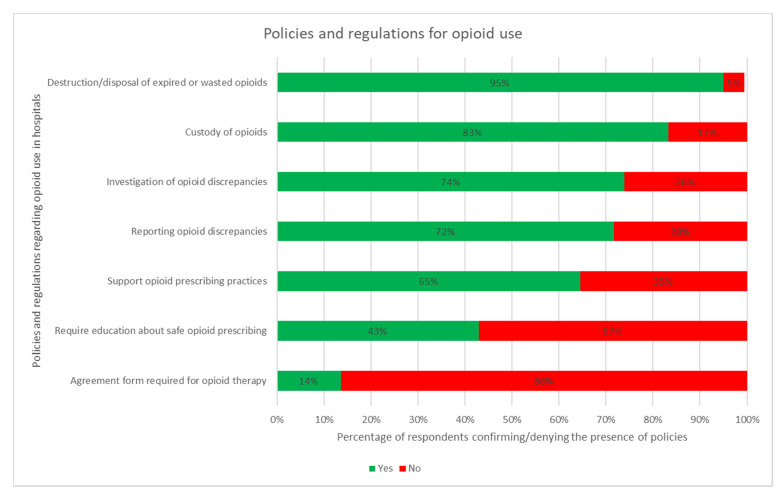
Proportion of hospitals included which implement the stated policies and regulations.

Upon questioning participants regarding policies and regulations at their hospital, it was determined that supporting opioid-prescribing practices, custody of opioids, destruction/disposal of expired or wasted opioids, and investigation of opioid discrepancies were common at most hospitals (Figure 1). Of the hospitals that did not have a policy for the investigation of opioid discrepancies, the majority also had no policy for the reporting of such discrepancies. A policy to educate providers regarding safe opioid prescribing was an uncommon policy. Furthermore, only six hospitals (14%) required patients to sign an agreement form for opioid therapy. A national legislation policy was followed by 33 (65%) hospitals. Of these hospitals, two also followed regional policies, five followed state legislation, and six also followed provincial legislation all of which were Canadian, indicating every Canadian hospital in the study follows both national and provincial legislation.

Regulation and policies for opioid use were similar across responses from different regions. Only two significant differences in regulation and policies by region were identified. This included hospital respondents from the Western Pacific Region being more likely to have regulations and/or guidelines to support opioid-prescribing practices at their place of practice (*p* = 0.009). In addition, hospital respondents from the American region were more likely to have a policy on reporting opioid discrepancies (*p* = 0.016).

As displayed in Figure 1, most participants (66%) agreed opioids are overused nationally; however, fewer agreed they are overused at their place of work. A similar trend was observed regarding the opioid crisis being a national problem; however, fewer participants agreed it is a problem at their place of work. Strong knowledge of opioids in the workplace was agreed to be an important aspect by most participants, and respondents agreed they would like further education on appropriate opioid use. Controversially, the belief that appropriate opioid use can cause addiction was a divisive issue.

**Figure 2:**
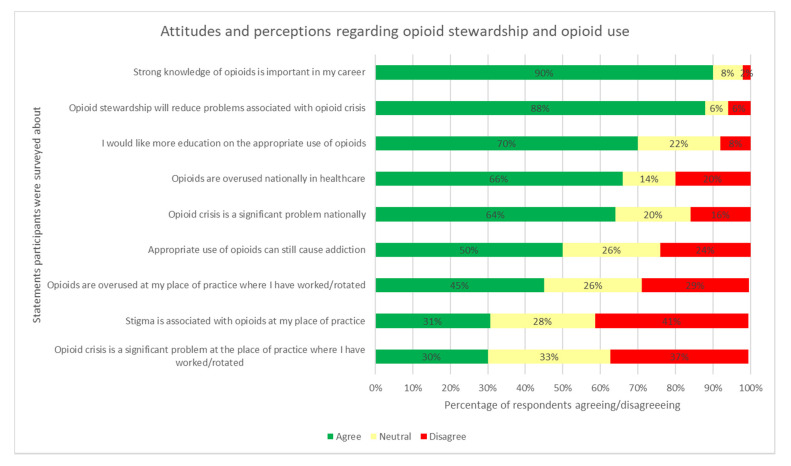
Proportion of participants’ agreeance with a selection of attitudes and perceptions regarding opioid stewardship and opioid use.

**Figure 3:**
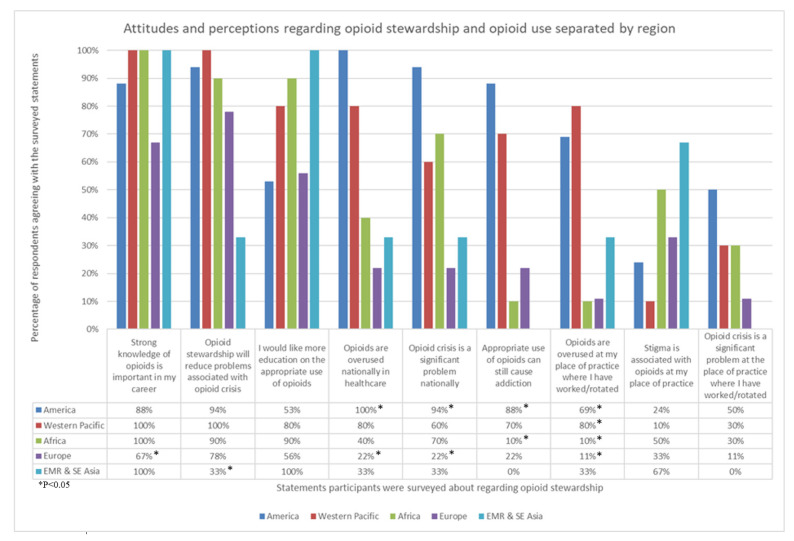
Proportion of participants agreeing with a selection of attitudes and perceptions regarding opioid stewardship and opioid use, separated by region.

The majority of participants (88%) agreed opioid stewardship would reduce problems associated with the opioid crisis (Figure 2); upon questioning participants about their perceptions regarding opioid stewardship practices, variable responses were observed among regions. As displayed in Figure 3, Europe was associated with disagreements regarding perceptions indicating the presence of opioid overuse. Countries in the American and Western Pacific Region mostly agreed on all statements, with disagreements regarding the presence of stigma surrounding opioid use at the participants’ place of practice. Conversely, countries surveyed in the EMR and SE Asia reported the highest prevalence (67%) of stigma associated with opioid use and were significantly less likely to agree that opioid stewardship would reduce problems associated with opioid overuse.

**Figure 4:**
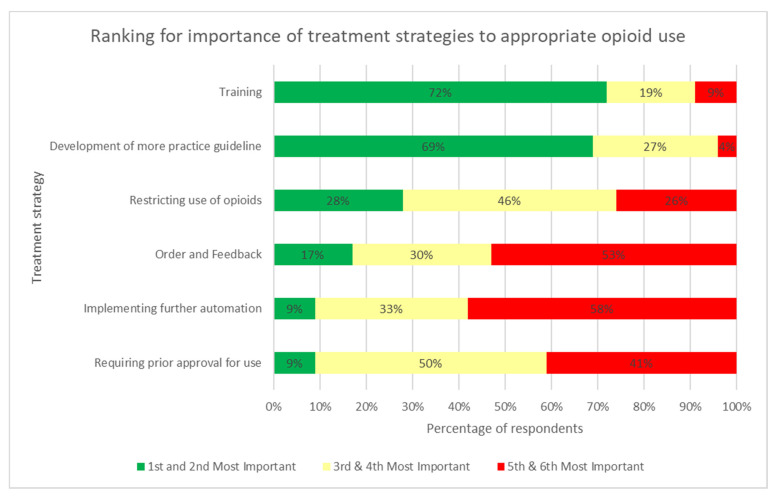
Participants’ rankings for the order of importance of treatment strategies to appropriate opioid use.

Figure 4 displays participants’ rankings in terms of the order of importance of treatment strategies to appropriate opioid use. Training was ranked as the most important practice, followed by the development of more practice guidelines, requiring prior approval for use, restricting opioid use, implementation of further automation, and order and feedback, respectively. Subsequently, participants were then asked to rank outcomes in order of importance. The outcome ranked to be most important by the majority of respondents was appropriate opioid use. Opioid-related mortality was ranked as the second most important outcome, and opioid-related length of stay was ranked as the third most important. Conversely, the opioid cost was determined to be the least important outcome by most respondents.

## 5. Discussion

This study showed that respondents from around the world generally utilise policies to support opioid-prescribing practices. Less common policies reported by respondents were the requirement for providers/caregivers to be educated about safe opioid prescribing and the requirement for patients to sign an agreement form for opioid therapy. Respondents reported a large variation in education required for prescribers surrounding opioids. Prescriber education and guidelines have been shown to successfully reduce the number of opioids initially prescribed by more than half [13]. This study showed that more than half of the respondents stated that their hospital did not have a policy for prescriber education, which was similar to a study conducted in the US where only 26.9% of hospitals responded having a policy that requires education of providers and/or caregivers about safe opioid prescribing [14]. Education of prescribers has been reported in Australia and New Zealand as one of the main opioid stewardship measures (78%) currently implemented. Furthermore, increased education on the use of opioid-free anesthesia and personalised opioid prescribing may have a large potential to reduce opioid use and its related adverse events [15,16,17]. Ensuring hospitals have a policy on prescriber education for safe opioid prescribing would assist in minimising such variations of opioid prescribing being observed around the world.

Respondents’ results indicate that they perceive the overuse of opioids as a greater issue in the American region and, to some extent, the Western Pacific region. Respondents from Australia, New Zealand, and the Philippines all agreed the opioid crisis and opioid overuse are national problems; however, respondents from Taiwan were the only Western Pacific country to disagree. This may be due to differences in cultural acceptance of opioid use where Asian countries often perceive opioid use negatively, reducing their use in Asian countries [18]. The overregulation of opioids in Asia, in addition to the heavy stigmatisation placed on opioid use, may also contribute to the use of opioids in Asian countries [19]. Physicians surveyed across 10 Asian countries have previously reported a reluctance to prescribe opioids due to fear of addiction (311/463, 67% agreed), fear of adverse events (301/463, 65% agreed), and hesitancy to report pain (243/463, 52% agreed), which are all patient-related barriers to optimal opioid therapy [20], indicating that the paucity of opioid use in Asian countries may be attributable to the associated stigma.

Conversely, respondents from European countries predominantly rejected the presence of an opioid crisis. A 2018 series of reports explored the implementation of drug policy programs in European countries, establishing that European approaches to battling the opioid crisis appear more efficacious through a variety of policies and support programs [21]. The European Monitoring Centre for Drugs and Drug Addiction reported only about 0.38% of the adult population of the EU and Norway were high-risk opioid users [22]. Furthermore, the United Kingdom (UK) appeared to have the highest rate of high-risk opioid users, at about 0.8%. The UK has reported it may be experiencing an opioid crisis, unlike other European countries, where opioids are involved in 9 out of 10 drug-induced deaths [23].

This study was not without limitations. As this study relied on participants completing a survey, there were numerous limitations. The survey had a small sample size, limiting the generalisability of the results. As the survey was disseminated during the rise of COVID-19, hospital pharmacists may have been unable to complete the survey due to such competing priorities. Additionally, as demographic characteristics of nonresponders were not available to the research team, no nonresponder analysis could be conducted. Furthermore, response bias is also a large contributing factor, as respondents’ personal views could impact the realistic depiction of the matter, or they may feel discouraged to provide what could be considered unfavourable answers causing their country or place of work to appear negatively.

## 6. Conclusions

In conclusion, healthcare professionals in the American region are more likely to perceive the opioid crisis as a problem, as opposed to those from the European region. Regardless of the presence or absence of a crisis, the implementation of further opioid education and stewardship practices are necessary globally and will contribute to safer prescribing and utilisation practices in hospitals.

## Figures and Tables

**Table 1:** Demographics of participants, including countries surveyed for each WHO region, and the number and percentage of respondents per country and region, hospital type, and prescribing methods.

Country of Origin		Number of Respondents n (%)
Africa		10 (20%)
•	Kenya	5 (10%)
•	Nigeria	5 (10%)
North and South America		17 (35%)
•	Canada	11 (22%)
•	Costa Rica	1 (2%)
•	United States	5 (10%)
Eastern Mediterranean and South East Asia		3 (6%)
•	Indonesia	2 (4%)
•	Iraq	1 (2%)
Europe		9 (18%)
•	France	3 (6%)
•	Germany	1 (2%)
•	Lithuania	1 (2%)
•	San Marino	1 (2%)
•	Serbia	1 (2%)
•	Spain	1 (2%)
•	United Kingdom	1 (2%)
Western Pacific		10 (20%)
•	Australia	2 (4%)
•	New Zealand	6 (12%)
•	Philippine	1 (2%)
•	Taiwan	1 (2%)
Type of hospital		
•	Academic hospital	25 (50%)
•	Community hospital	16 (32%)
•	Unspecified	9 (18%)
Prescribing system		
•	Computerised	23 (46%)
•	Paper-based	23 (46%)
•	Hybrid of paper and computerised	2 (4%)
Respondent gender		
•	Male	15 (30%)
•	Female	34 (68%)
Type of health professional		
•	Pharmacist	47 (94%)

## Data Availability

The data presented in this study are available on request from the corresponding author. The data are not publicly available as the ethics committee has not approved the data to be made available.

## Appendix A

Images of thesurvey distributed to the International Pharmaceutical Federation for completion:

## References

[1] Hall K.K., Shoemaker-Hunt S., Hoffman L., Richard S., Gall E., Schoyer E., Costar D., Gale B., Schiff G., Miller K. (2020). Making Healthcare Safer III: A Critical Analysis of Existing and Emerging Patient Safety Practices.

[2] World Health Organization (2020). Opioid Overdose. https://www.who.int/news-room/fact-sheets/detail/opioid-overdose.

[3] Shah A., Hayes C.J., Martin B.C. (2017). Factors Influencing Long-Term Opioid Use among Opioid Naive Patients: An Examination of Initial Prescription Characteristics and Pain Etiologies. J. Pain.

[4] Herzig S.J., Rothberg M.B., Cheung M., Ngo L.H., Marcantonio E.R. (2013). Opioid utilization and opioid-related adverse events in nonsurgical patients in US hospitals. J. Hosp. Med..

[5] Calcaterra S.L., Yamashita T.E., Min S.-J., Keniston A., Frank J.W., Binswanger I.A. (2016). Opioid Prescribing at Hospital Discharge Contributes to Chronic Opioid Use. J. Gen. Intern. Med..

[6] Danovitch I., VanLe B., Van Groningen N., Ishak W., Nuckols T. (2020). Opioid Overdose in the Hospital Setting: A Systematic Review. J. Addict. Med..

[7] Australian Commission on Safety and Quality in Health Care (2021). National Opioid Analgesic Stewardship Program—Discussion Paper.

[8] Liu S., Gnjidic D., Nguyen J., Penm J. (2019). Effectiveness of interventions on the appropriate use of opioids for noncancer pain among hospital inpatients: A systematic review. Br. J. Clin. Pharmacol..

[9] A Pedersen C., Schneider P.J., Ganio M.C., Scheckelhoff D.J. (2020). ASHP national survey of pharmacy practice in hospital settings: Prescribing and transcribing—2019. Am. J. Health Pharm..

[10] Allen M.L., Leslie K., Parker A.V., Kim C.C., Brooks S.L., Braat S., Schug S.A., Story D.A. (2019). Post-surgical opioid stewardship programs across Australia and New Zealand: Current situation and future decisions. Anaesth. Intensive Care.

[11] Harris P.A., Taylor R., Thielke R., Payne J., Gonzalez N., Conde J.G. (2009). Research electronic data capture (REDCap)—A metadata-driven methodology and workflow process for providing translational research informatics support. J. Biomed. Inform..

[12] World Health Organization (2020). Working with the Regions. https://www.who.int/chp/about/regions/en/.

[13] Hill M.V., Stucke R.S., McMahon M.L., Beeman J.L., Barth R.J. (2018). An Educational Intervention Decreases Opioid Prescribing After General Surgical Operations. Ann. Surg..

[14] Phelps P., Achey T.S., Mieure K.D., Cuellar L., MacMaster H., Pecho R., Ghafoor V. (2018). A Survey of Opioid Medication Stewardship Practices at Academic Medical Centers. Hosp. Pharm..

[15] Bugada D., Lorini L.F., Fumagalli R., Allegri M. (2020). Genetics and Opioids: Towards More Appropriate Prescription in Cancer Pain. Cancers.

[16] Manchikanti L., Kaye A.M., Knezevic N.N., McAnally H., Slavin K., Trescot A.M., Blank S., Pampati V., Abdi S., Grider J.S. (2017). Responsible, Safe, and Effective Prescription of Opioids for Chronic Non-Cancer Pain: American Society of Interventional Pain Physicians (ASIPP) Guidelines. Pain Physician.

[17] Bugada D., Lorini L.F., Lavand’Homme P. (2020). Opioid Free Anesthesia: Evidence for Short and Long-Term Outcome.

[18] Pardo B., Kilmer B., Huang W. (2019). Contemporary Asian Drug Policy: Insights and Opportunities for Change. Contemporary Asian Drug Policy: Insights and Opportunities for Change.

[19] Cleary J., Radbruch L., Torode J., Cherny N.I. (2013). Formulary availability and regulatory barriers to accessibility of opioids for cancer pain in Asia: A report from the Global Opioid Policy Initiative (GOPI). Ann. Oncol..

[20] Kim Y., Ahn J.S., Calimag M.M.P., Chao T.C., Ho K.Y., Tho L.M., Xia Z., Ward L., Moon H., The ACHEON Working Group (2015). Current practices in cancer pain management in Asia: A survey of patients and physicians across 10 countries. Cancer Med..

[21] North Carolina Health News (2020). Lessons from Abroad: How Europeans Have Tackled Opioid Addiction and What the U.S. Could Learn from Them, in North Carolina Health News.

[22] European Monitoring Centre for Drugs and Drug Addiction (2017). European Drug Report: Trends and Developments.

[23] European Monitoring Centre for Drugs and Drug Addiction (2019). United Kingdom Country Drug Report 2019.

